# Essential role of cytochrome *bc*_1_ in *Pseudomonas aeruginosa* cell physiology and virulence

**DOI:** 10.3389/fcimb.2026.1763669

**Published:** 2026-02-25

**Authors:** Jennifer M. Sorescu, Martín A. González-Montalvo, Nancy Patel, Gabriella Baltes, Shriya R. Avula, Karina Tuz, Oscar X. Juárez

**Affiliations:** Department of Biological Sciences, Illinois Institute of Technology, Chicago, IL, United States

**Keywords:** antibiotic resistance, antibiotics, cytochrome *bc*_1_, electron transport, *Pseudomonas aeruginosa*, respiratory chain, type III secretion system (T3SS), virulence factor

## Abstract

*Pseudomonas aeruginosa* is a multidrug-resistant Gram-negative nosocomial pathogen posing a major healthcare burden due to its intrinsic antibiotic resistance and environmental adaptability, which continue to significantly constrain therapeutic options. This pathogen is a leading cause of hospital-acquired respiratory and urinary tract infections that are often persistent and difficult to treat. The elucidation of metabolic adaptations permitting *P. aeruginosa* survival and supporting virulence in diverse host environments is essential for informing novel treatments. This study investigates the role of cytochrome *bc*_1_ in *P. aeruginosa* PA14 growth, energetics, and virulence *in vitro*, under physiologically relevant conditions, and *in vivo*. The *bc*_1_ deletion mutant exhibits significant growth defects in urinary- and lung-like media, demonstrating its importance in PA14 physiology, and displays reduced virulence in bladder epithelial cell monolayers, mediated by decreased type III secretion system (T3SS) toxin expression. *In vivo*, the mutant was nearly avirulent in a murine systemic infection model. Bioenergetic analysis revealed severe decline in PA14 Δ*bc*_1_ ATP production, aligning with the enzyme’s central role in respiration and providing a mechanistic explanation for the mutant’s reduced T3SS function, as toxin secretion requires energy. This work provides compelling evidence of direct T3SS regulation by cytochrome *bc*_1_, revealing a previously unrecognized link between respiratory metabolism and virulence. Metabolic profiling indicates that to compensate for cytochrome *bc*_1_ loss, the mutant relies on NDH-2 and *bd* oxidase, permitting cellular survival but substantially reducing ATP yield. Collectively, these findings establish cytochrome *bc*_1_ as a target for antibiotic development against *P. aeruginosa*, impacting bioenergetics, physiology, and virulence.

## Introduction

Multidrug-resistant *Pseudomonas aeruginosa* is an opportunistic pathogen that poses a serious threat to human health due to its prevalence and lack of effective therapeutic treatments. In 2019, *P. aeruginosa* was the sixth leading global pathogen in deaths associated with antimicrobial resistance (Murray et al., 2022). This problem has become more challenging, as multidrug-resistant *P. aeruginosa* infections in hospital-onset cases increased >30% in only two years (2019-2020) (COVID-19 Special Report, 2022). The World Health Organization (WHO) designated carbapenem-resistant *P. aeruginosa* as a high priority pathogen, emphasizing the urgent need for development of new antibiotics against this bacterium (WHO Bacterial Priority Pathogens List 2024, 2024). *P. aeruginosa* accounts for approximately 23% of ICU-acquired infections worldwide and 7% of healthcare-associated infections in the U.S., causing acute and chronic infections in immunocompromised patients, particularly those suffering from respiratory diseases and urinary tract infections (UTIs) (Qin et al., 2022; Reynolds and Kollef, 2021). *P. aeruginosa* is responsible for 7-10% of all UTIs and 10% of catheter-associated UTIs (CAUTIs) (Mancuso et al., 2023; Reynolds and Kollef, 2021). Although uropathogenic *Escherichia coli* has a higher prevalence (65%) among nosocomial UTIs (Mancuso et al., 2023; Newman et al., 2022), infections caused by *P. aeruginosa* are considerably more difficult to treat due to their increased propensity for antibiotic resistance. Approximately 10% of *P. aeruginosa* isolates from healthcare settings are multidrug-resistant, with over 10% of CAUTIs caused by multidrug-resistant (MDR) strains (Multidrug-resistant Pseudomonas aeruginosa, 2022). Moreover, *P. aeruginosa* is the second most prevalent cause of ventilator-associated pneumonia, responsible for 26% of cases globally (Reynolds and Kollef, 2021). Additionally, *P. aeruginosa* is the predominant pathogen in chronic cystic fibrosis (CF) cases, found in 40-50% of adult cases (Nickerson et al., 2024), which increases morbidity and mortality (Chmiel and Davis, 2003; Kamal et al., 2024; Lyczak et al., 2000). Similarly, the chronic presence of *P. aeruginosa* in respiratory secretions is a major predictor of morbidity and mortality in children with CF (Rosenfeld et al., 2001). Chronic colonization of the respiratory tract fosters an increase in mutations, adaptations, and persistence that contribute to treatment resistance (Moradali et al., 2017). It is evident that the development of novel antibiotics is critical to reduce the impact of this pathogen on global healthcare outcomes. *P. aeruginosa* infections are treated with antibiotics such as penicillins, cephalosporins, fluoroquinolones, aminoglycosides, or carbapenems (Kunz Coyne et al., 2022). However, *P. aeruginosa* produces drug-tolerant persister cells that are prevalent in biofilms (Narten et al., 2012; Ciofu et al., 2017; Soares et al., 2020), which reduce treatment efficacy and give rise to MDR, extensively drug-resistant (XDR), or pan drug-resistant (PDR) infections (Eid et al., 2025). There are limited therapeutic options for managing such infections, and inappropriate initial treatment or delays in administering effective therapy often result in treatment failure (Bassetti et al., 2020). Thus, there is an urgent need for identification of novel antibiotic targets that can be exploited against *P. aeruginosa*.

The respiratory chain of *P. aeruginosa* is highly branched, allowing adaptation to a multitude of environments due to its unique flexibility (Stover et al., 2000; Williams et al., 2007; Mikkelsen et al., 2011). In aerobic conditions, the bacterium primarily relies on oxidative phosphorylation to drive ATP production (Williams et al., 2007; Alvarez-Ortega and Harwood, 2007; Arai, 2011) rather than fermentation (Williams et al., 2007; Arai, 2011; La Rosa et al., 2018, 2019; Alvarez-Ortega and Harwood, 2007), utilizing 17 dehydrogenases (Stover et al., 2000) that facilitate electron transfer to either ubiquinone, nitrate reductases, or cytochrome *c* (Stover et al., 2000; Williams et al., 2007). Electrons are subsequently funneled to oxygen from quinol via cytochrome *bo*_3_ oxidase, cyanide-insensitive *bd*-like oxidase, or cytochrome *bc*_1_ and cytochrome *c* oxidases (Arai, 2011; Giuffrè et al., 2014; Stover et al., 2000; Williams et al., 2007). Our group previously investigated the respiratory metabolism of *P. aeruginosa* in urine-like conditions (Liang et al., 2020). We reported that cytochrome *bc*_1_ is a main component of the pathogen’s respiratory chain in both logarithmic and stationary growth phases, accounting for 50-60% of the respiratory activity (Hu et al., 2024), indicating that this could be an important pharmacological target. Cytochrome *bc*_1_ has been effectively exploited as a therapeutic target in parasitic and fungal pathogens by the FDA-approved drug atovaquone, underscoring substantial structural divergence between pathogenic and mammalian complexes and supporting its viability as an effective and selective drug target (Baggish and Hill, 2002; Walker et al., 1998).

Given the clinical importance of *P. aeruginosa* and the growing need for novel antimicrobial strategies, we sought to explore the functional role of cytochrome *bc*_1_ in the reference strain PA14, a highly virulent strain isolated from a burn wound (He et al., 2004; Wiehlmann et al., 2007), characterizing the role of this critical enzyme in bacterial bioenergetics, homeostasis, and virulence. Our data show that the deletion of cytochrome *bc*_1_ produced significant defects in bacterial proliferation in LB and the physiologically relevant modified artificial urinary medium (mAUM) and lung medium (LM). Moreover, the mutant exhibited reduced virulence in an *in vitro* infection model of human bladder cells, which was due to a decrease in the expression of the type 3 secretion system (T3SS), and was reflected in an almost completely avirulent phenotype in a murine model. A significant reduction in ATP production was observed in the mutant during logarithmic and stationary growth phases across all media tested, which provides a direct link to the diminished function of the T3SS, driven by ATP hydrolysis. Analysis of the metabolic adaptations the pathogen undergoes to compensate for loss of the enzyme indicates a reliance on the type-2 NADH dehydrogenase (NDH-2) and the cyanide-insensitive *bd* oxidase, which allows the bacteria to survive the elimination of this critical enzyme, but significantly decreases its ability to produce ATP and, consequently, to cause disease. These findings validate cytochrome *bc*_1_ as a pharmacological target with significant potential for therapeutic development against *P. aeruginosa*.

## Materials and methods

### Mutant construction

To generate the PA14 Δ*bc*_1_ mutant, a lambda-red recombinase method was used (Bru et al., 2019; Datsenko and Wanner, 2000). *P. aeruginosa* PA14 cells were transformed with pUCP18-RedS as previously described (Lesic and Rahme, 2008) and selected in LB medium + carbenicillin 300 µg/mL. The lambda-red system in cells harboring pUCP18-RedS was induced with 10 mM arabinose. Cells were pelleted at 16,000 × *g* for 10 minutes at room temperature and were washed with 300 mM sucrose. Electrocompetent cells were transformed with the mutagenic amplicon into pUCP18-RedS-positive cells in a 0.4 cm gap cuvette at 2500 V using a MicroPulser Electroporator (Bio-Rad, Hercules, CA) as previously reported (Bru et al., 2019). For the mutagenic amplicon, regions of 500–650 bp upstream and downstream of the operon encoding cytochrome *bc*_1_ (*petA*-*cytB*-*cytC*_1_) were amplified using the primer pairs listed in **Table 1**. The gentamicin cassette of pAS03 was amplified using the primers Gm_pAS03_F and Gm_pAS03_R (**Table 1**). The three amplicons were combined and amplified via fusion PCR using the *Bc*_1_ Upstream Bc1_Mut_F and *Bc*_1_ Downstream Bc1_Mut_R primers. Mutants were selected on gentamicin plates (30 µg/mL) and verified by PCR using the primers Bc1_F and Bc1_R and by nanopore bacterial genomic sequencing (Eurofins Genomics, Louisville, KY). Our sequencing data show a single elimination of the *bc*_1_ operon with no insertions or deletions in any other site. The genome sequence of the mutant is available upon request.

**Table 1:** Primers for mutant construction.

Primer pair	Primer	Sequence (5’-3’)
Gm cassette	Gm_pAS03_F	TGTGTAGGCTGGAGCTGCTTCGAAG
	Gm_pAS03_R	ATTCCGGGGATCCGTCG
*Bc*_1_ Upstream	Bc1_Mut_F	CGTGCCCTGATCGAGTACGAC
	Bc1_Mut_R	CCAGCTGCCTAGGGGCCTTTCTTGCCCACGTTCACCTGCAC
*Bc*_1_ Downstream	Bc1_Mut_F	GAAGCAGCTCCAGCCTACACAATCTACGCCCGGGAAAACGGC
	Bc1_Mut_R	CGCCAAGGCCGATCCAGCCC
Verification	Bc1_F	CTGACAGAGCTCGTAAAGGAGGAATTAACCATGAGTAATGACGGCG
	Bc1_R	GTCACTCAGCTGTTACTTGACCACCTTCAGGGATGG

### *P. aeruginosa* growth and analysis

*P. aeruginosa* strains PA14 (wild-type) and PA14 Δ*bc*_1_ were grown in LB Broth (Miller), modified artificial urinary medium (mAUM), and lung medium (LM) at 37°C with shaking at 250 RPM as reported previously (Hu et al., 2024; Liang et al., 2020; Ruhluel et al., 2022). mAUM and LM were prepared as described previously (Hu et al., 2024; Liang et al., 2020; Ruhluel et al., 2022). Growth was assessed by following the absorbance of cell cultures at 620 nm using an 800 TS microplate reader (BioTek, Winooski, VT). Maximum growth, duplication time, and lag phase duration were calculated by fitting the growth data to logistic functions, as previously detailed in our work (Liang et al., 2020; Kahm et al., 2010). Experiments were conducted in triplicate, with results reported as mean ± SD, *n* = 3. Statistical significance between PA14 and PA14 Δ*bc*_1_ was assessed using *t*-test analysis.

### Human urinary bladder 5637 carcinoma cell infection assay

5637 human urinary bladder carcinoma cells (ATCC HTB-9) were cultured in RPMI 1640 medium supplemented with 10% Fetal Bovine Serum (FBS). Following treatment with 0.25% trypsin-EDTA, 2.0 x 10^5^ cells were seeded in 24 well plates and incubated for 48 hours at 37 °C with 5% CO_2_ in a humidified atmosphere prior to infection. *P. aeruginosa* strains PA14 and PA14 Δ*bc*_1_ were grown for 24 hours in LB at 37 °C under continuous agitation (250 RPM). Bacterial cells were centrifuged at 16,000 × *g* for 10 minutes at room temperature and washed with PBS. Monolayers of 5637 cells were washed with PBS, and fresh RPMI 1640 supplemented with 10% FBS was added. Bladder cells were then infected with PA14 or PA14 Δ*bc*_1_ at a MOI of 10 for 2, 4, and 6 hours. At each time point, cells were washed with PBS and stained with a 1:1 mixture of PBS:trypan blue (0.4%). Bright-field images were collected using a Keyence BZ-X710 Fluorescence Microscope (Itasca, IL) with a cooled monochrome CCD camera and Nikon Plan Fluor ELWD 20x/0.45 Ph1 ADM, ∞/0–2 WD 8.2–6.9 lens. Quantification of cell viability post-infection was performed using the BZ-X analyzer (version 01.03.00.05) and ImageJ v1.54p (Abramoff et al., 2004), with assessors blinded to outcomes. Viability was quantified by calculating the percentage of live cells (unstained) from the total cells [unstained plus stained (dead)]. Results are reported as mean ± SD, *n* = 3, with statistical significance between PA14 and PA14 Δ*bc*_1_ assessed via *t*-test.

### Type III secretion system detection

Following 5637 cell infection with *P. aeruginosa* strains PA14 or PA14 Δ*bc*_1_, expression of the type III secretion system (T3SS) proteins exoT, exoU, and exoY was assessed in the media of the infected cell culture at 4 hours post-infection (hpi). Supernatant total RNA was extracted using TRIzol LS (Thermo Fisher Scientific, Waltham, MA) and quantified on a Qubit 3.0 fluorometer using the AccuBlue Broad Range RNA Quantitation Kit (Biotium, Fremont, CA), followed by cDNA synthesis with the ProtoScript II First Strand cDNA Synthesis Kit using the Random Primer Mix (New England Biolabs, Ipswich, MA). qPCR was subsequently performed using the primer pairs listed in **Table 2**, designed using Primer 3 software (Koressaar and Remm, 2007; Untergasser et al., 2012; Kõressaar et al., 2018). *P. aeruginosa rpoD* was utilized as a housekeeping gene due to its vital role in promoter identification to facilitate constitutive gene expression (Potvin et al., 2008; Savli et al., 2003). cDNA (100 ng) amplification and quantification were performed on a Bio-Rad CFX Connect Real-Time PCR Detection System using the iTaq Universal SYBR Green Supermix kit (Bio-Rad, Hercules, CA). mRNA expression was determined via a 2-ΔΔCT method comparing *rpoD*, *exoT*, *exoU*, and *exoY* expression in the presence of *P. aeruginosa* PA14 or PA14 Δ*bc*_1_ infection via cycle threshold analysis (Livak and Schmittgen, 2001). Data are presented as mean ± SE, *n* = 9, with statistical significance determined via *t*-test.

**Table 2:** Primers for T3SS Detection.

Target gene	Sequence (5’-3’)	Amplicon size (bp)
*exoT*	TGCATGCGGTAATGGACAAG GTATAGAGACCGAGCGCCAT	190
*exoU*	CGTACTCTGACTGACTCGGC GCCTTTCTCTTCTAGCGCCA	171
*exoY*	ATGACCGCCGATTATGACCT CTCCCTGCCATAGAATCCGT	150
*rpoD*	GCATCCTGGCCGACTACAAT TGCTGTCGTCGCTTTCTTCT	183

### Mouse model of *P. aeruginosa* infection

*P. aeruginosa* infection in mice was performed using a sepsis model, as described previously (Six et al., 2021). PA14 and PA14 Δ*bc*_1_ were grown in LB medium for 24 hours, followed by centrifugation at 16,000 × *g* for 10 minutes at room temperature. Cells were washed with PBS and eight-week-old female BALB/cJ mice (The Jackson Laboratory, Bar Harbor, ME) were inoculated with 50 µl of PBS (*n* = 7), 1.25-1.64 x 10^7^ CFUs of PA14 (*n* = 17), or 1.50-1.60 x 10^7^ CFUs of PA14 Δ*bc*_1_ (*n* = 14) intravenously through lateral tail vein using a 29G syringe, according to Illinois Institute of Technology IACUC-approved protocols. Animals were provided with unrestricted access to food and water and kept on a standard 12 h light/dark cycle with lights off at 7:00 p.m. Animals’ weights and survival were monitored twice daily for one week post-infection. Survival outcomes were visualized with Kaplan-Meier curves and statistically evaluated using the log-rank test implemented in OriginLab (version Origin 2025b (10.25), OriginLab Corporation, Northampton, MA).

### Biofilm determination

Biofilm formation was assessed for *P. aeruginosa* strains PA14 and PA14 Δ*bc*_1_, as described previously (O’Toole, 2011). Bacterial overnight cultures were grown in LB at 37 °C with shaking (250 RPM). One milliliter of bacteria culture was centrifuged at 16,000 × *g* for 10 minutes at room temperature, and cells were washed with PBS twice. Bacterial pellets were resuspended in 1 mL of PBS and adjusted to OD_600_ of 1.0. This suspension was further diluted to an OD_600_ of 0.01 in LB, mAUM, or LM. 100 uL of each suspension was transferred to individual wells of 96-well plates. Experiments were performed three times using 10 technical replicates per condition and strain. Plates were incubated at 37 °C for 48 hours without shaking. Following incubation, supernatant was removed, and wells were washed three times with PBS and allowed to air dry. Biofilm was stained with 1% filtered crystal violet for 30 minutes, dye was removed, and wells were washed with ultrapure water. Retained crystal violet was solubilized with 33% acetic acid and absorbance was read at 595 nm using an 800 TS microplate reader (BioTek, Winooski, VT). Statistical significance between PA14 and PA14 Δ*bc*_1_ was determined via *t*-test.

### ATP quantification

ATP detection was performed using the BacTiter-Glo™ Microbial Cell Viability Assay (Promega, Madison, WI) according to manufacturer’s instructions. Cultures of *P. aeruginosa* strains PA14 and PA14 Δ*bc*_1_ harvested during both logarithmic and stationary phases following growth in LB, mAUM, and LM were vortexed and combined with BacTiter-Glo™ reagent in a 1:1 ratio in 96-well white opaque-walled plates. For cultures grown in LB, mAUM, and LM, logarithmic growth was defined at 7, 7, and 16 hours post-inoculation, respectively, whereas stationary phase was designated at 24, 12, and 24 hours for LB, mAUM, and LM, respectively. Culture reagent mixtures were incubated for five minutes and luminescence was recorded. ATP standard curves were prepared in LB, mAUM, and LM and obtained values were interpolated based on the appropriate media to obtain moles of ATP in each sample. Protein was quantified via BCA assay (Thermo Fisher Scientific, cat. no. 23225, Waltham, MA) and ATP quantity was subsequently normalized to protein concentration. Experiment was performed in triplicate, with results reported as mean ± SD, *n* = 3. Statistical significance between PA14 and PA14 Δ*bc*_1_ was determined using a *t*-test.

### Reactive oxygen species detection

ROS detection was carried out according to Fraser-Pitt et al. (Fraser-Pitt et al., 2018). *P. aeruginosa* strains PA14 and PA14 Δ*bc*_1_ were grown in LB, mAUM, and LM at 37 °C with shaking at 250 RPM and harvested in the logarithmic and stationary phases of growth. Samples were subsequently centrifuged at 16,000 × *g* for 10 minutes and washed with PBS at room temperature. H_2_DCFDA (2’,7’-dichlorodihydrofluorescein diacetate) (Invitrogen, cat. no. D399, Waltham, MA) was added to 100 µL cell samples at a concentration of 1 µM in a 96-well black clear bottom plate and fluorescence was assessed at 480/520 nm excitation/emission using a SpectraMax iD5 Multi-Mode Microplate Reader (Molecular Devices, San Jose, CA). Experiments were performed in triplicate. Data were normalized to sample protein concentration, quantified via BCA assay, and reported as relative light units (RLU)/µg protein. Statistical significance was determined via *t*-test analysis.

### Determination of membrane potential

Membrane potential was assessed with JC-1 dye (Invitrogen, cat. no. T3168, Waltham, MA) in *P. aeruginosa* strains PA14 and PA14 Δ*bc*_1_ harvested during both logarithmic and stationary phases of growth in LB, mAUM, and LM. JC-1 (10 µM) was added to harvested bacteria (2.5 µg of protein) in a 96-well black clear bottom plate and incubated for 10 minutes at room temperature in the dark, with or without oligomycin (1 µM) and carbonyl cyanide m-chlorophenyl hydrazone (CCCP) (5 µM). The conditions in which membrane potential formation were analyzed include basal metabolic state (control), presence of oligomycin (1 µM) (Symersky et al., 2012) to induce hyperpolarization by inhibiting ATP synthesis, and the presence of the protonophore CCCP (5 µM) (Daniels et al., 1981) to depolarize the membrane. Fluorescence was measured at 480/520 and 535/596 nm excitation/emission for the green and red fluorescent states of the dye. Results were presented as the ratio of red to green fluorescence. Cell autofluorescence (red and green) was subtracted for all samples. After conducting the experiment in triplicate, the data were analyzed using *t*-test analysis.

### Membrane preparation

Membranes were obtained from *P. aeruginosa* PA14 Δ*bc*_1_ grown in mAUM during the logarithmic stage of growth, as previously described (Hu et al., 2024; Liang et al., 2020). Cell pellets were washed with KHE buffer (150 mM KCl, 20 mM HEPES, 1 mM EDTA, pH 7.5) and stored at -80 °C until further use. Bacterial cells were thawed and resuspended in KHE buffer with 5 mM MgCl_2_, 10 µg/mL DNase, and 1 mM PMSF. Cells were ruptured at 16,000 PSI using an Avestin Emulsiflex-C5 high pressure homogenizer (Ottawa, ON, Canada). Debris was separated from membranes via centrifugation at 10,000 × *g* (4 °C) and cell membranes were subsequently collected from supernatant via ultracentrifugation at 100,000 × *g* (4 °C). Membrane fractions were washed with KHE buffer and stored at -80 °C.

### Respiratory activity

The respiratory activity of *P. aeruginosa* PA14 Δ*bc*_1_ membranes harvested in the logarithmic phase following growth in mAUM was measured in a customized 1.6 mL glass oximetric chamber using a Clark-type oxygen electrode (YSI 5300) at 37 °C using the methods previously detailed (Hu et al., 2024; Liang et al., 2020) in triplicate. Assays were performed in KHE buffer with and without 1 µM rotenone to inhibit complex I, with 200 µM NADH or deamino-NADH to test for NADH oxidase activity, 10 mM lactate to test for lactate dehydrogenase activity, and 1 mM succinate to test for succinate dehydrogenase activity. Ubiquinol oxidase activity was determined using 50 µM ubiquinone-1, 500 µM 1,4-Dithiothreitol (DTT), and various concentrations of KCN to inhibit terminal oxidases.

## Results

### Cytochrome *bc*_1_ is critical for growth in physiologically relevant medium

**Figure 1:**
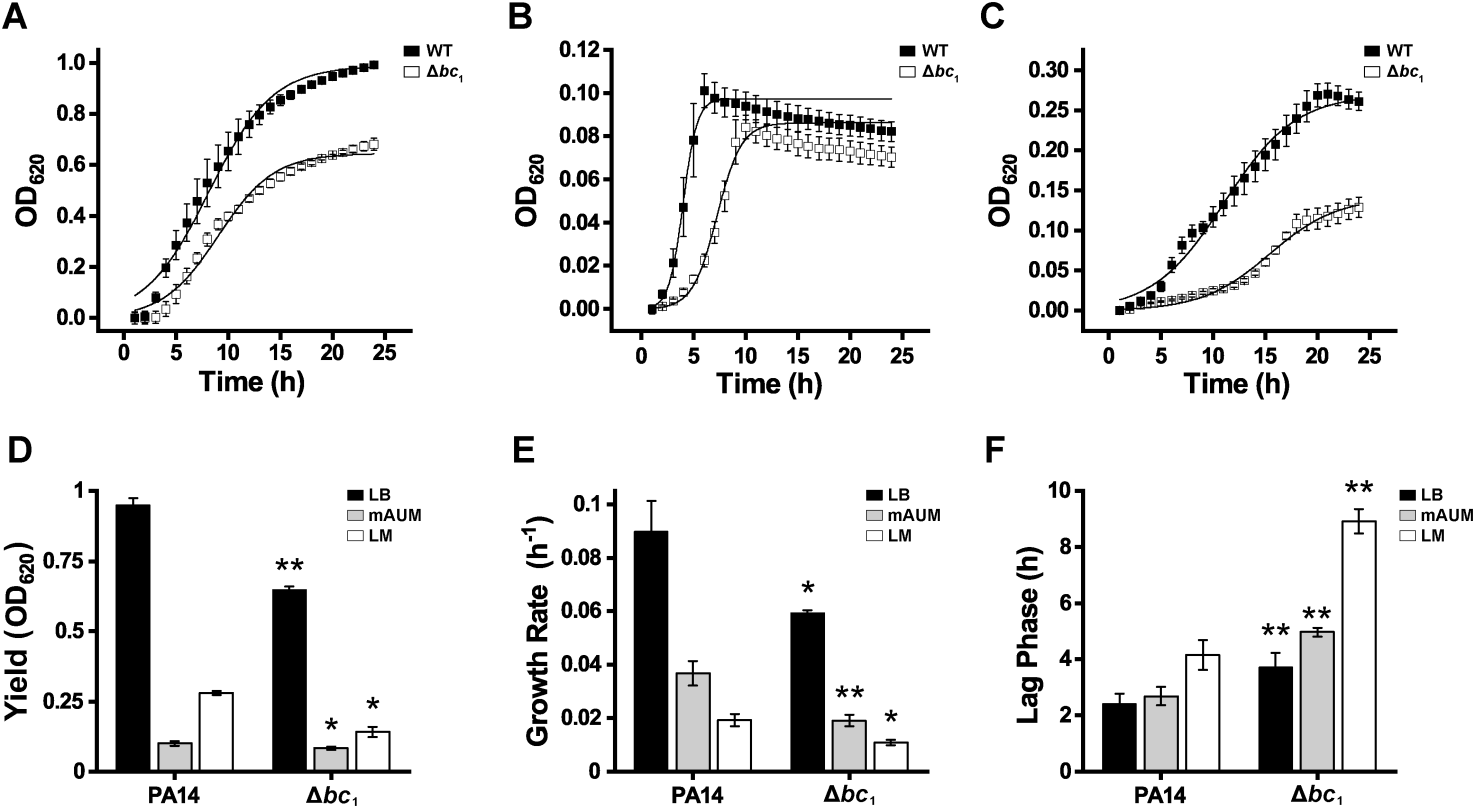
Growth of PA14 and PA14 Δ*bc*_1_ in physiologically relevant medium. PA14 and PA14 Δ*bc*_1_ growth (24 h) in LB **(A)**, mAUM **(B)**, and LM **(C)**. Growth parameter analysis shows maximum growth **(D)**, growth rate **(E)**, and lag phase duration **(F)** of PA14 and PA14 Δ*bc*_1_ grown in physiologically relevant medium. Results are presented as mean ± SD, *n* = 3. Asterisks denote significant differences between PA14 and PA14 Δ*bc*_1_ based on *t*-test analyses (*p < 0.05, **p < 0.01).

As reported in our previous work, cytochrome *bc*_1_ is critical for aerobic metabolism, ATP synthesis, and ion pumping in *P. aeruginosa* (Hu et al., 2024; Liang et al., 2020). To assess the role of this enzyme in supporting proliferation under physiologically relevant conditions, growth of wild-type PA14 and the deletion mutant was assessed in media resembling the environment of common sites of infection, such as the urinary tract (mAUM) and the respiratory epithelium (LM), and compared to growth in nutrient-rich laboratory LB medium (**Figure 1**). Previous work was carried out in a PA14 Δ*bc*_1_ transposon mutant (Hu et al., 2024), which was not completely characterized at the genomic level, and may carry additional genetic modifications. In this work, we characterized the PA14 strain in which the *bc_1_* gene was specifically eliminated using the lamba-red recombination system. Our group has previously validated mAUM for the analysis of adaptations in urine-like conditions (Liang et al., 2020), while LM formulation has been used to study the growth of other pathogens in synthetic lung media (Ashworth et al., 2024; Ruhluel et al., 2022). Determining *P. aeruginosa* growth in urine-like medium and lung medium is critical for understanding how this pathogen adapts to the unique nutrient conditions of these environments that mimic infection sites, which can inform treatment strategies for chronic and acute urinary and respiratory infections such as (CA)UTIs and cystic fibrosis infections.

The growth of the wild-type and Δ*bc*_1_ mutant was followed in LB (**Figure 1A**), mAUM (**Figure 1B**), and LM (**Figure 1C**). In LB, the mutant strain exhibited significantly lower growth and reached a maximum OD_620_ 32% lower than the wild-type (**Figure 1D**). Moreover, duplication time was reduced by 33% in the mutant (**Figure 1E**) and the lag phase duration was increased 1.5-fold (**Figure 1F**). These results confirm the important role of the *bc*_1_ complex in this growth condition, as shown from our previous work (Hu et al., 2024). Similarly, in urine-like conditions (mAUM), maximal bacterial growth was decreased in the mutant, approximately 16% (**Figures 1B, D**). Moreover, the duplication rate was nearly half compared to wild-type strain (**Figure 1E**), while its lag phase was almost doubled (**Figure 1F**). The most pronounced difference in growth between the wild-type and the mutant is observed in LM (**Figure 1C**). The mutant had a 50% reduction in total growth (**Figure 1D**), more than doubling of the lag phase (**Figure 1F**), and a reduction in the growth rate >50% (**Figure 1E**). The behavior exhibited by the mutant in these conditions suggests a hindered capacity to produce energy under physiologically relevant environments. This impairment may be due to the disruption of the electron transport chain in the mutant, highlighting the essential role of cytochrome *bc*_1_ in maintaining energy metabolism across diverse conditions. Collectively, these findings emphasize the importance of cytochrome *bc*_1_ as a key respiratory component that enables *P. aeruginosa* to adapt and proliferate in both urinary environments and nutrient-limited lung conditions.

### Cytochrome *bc*_1_ is critical for virulence in *P. aeruginosa*

**Figure 2:**
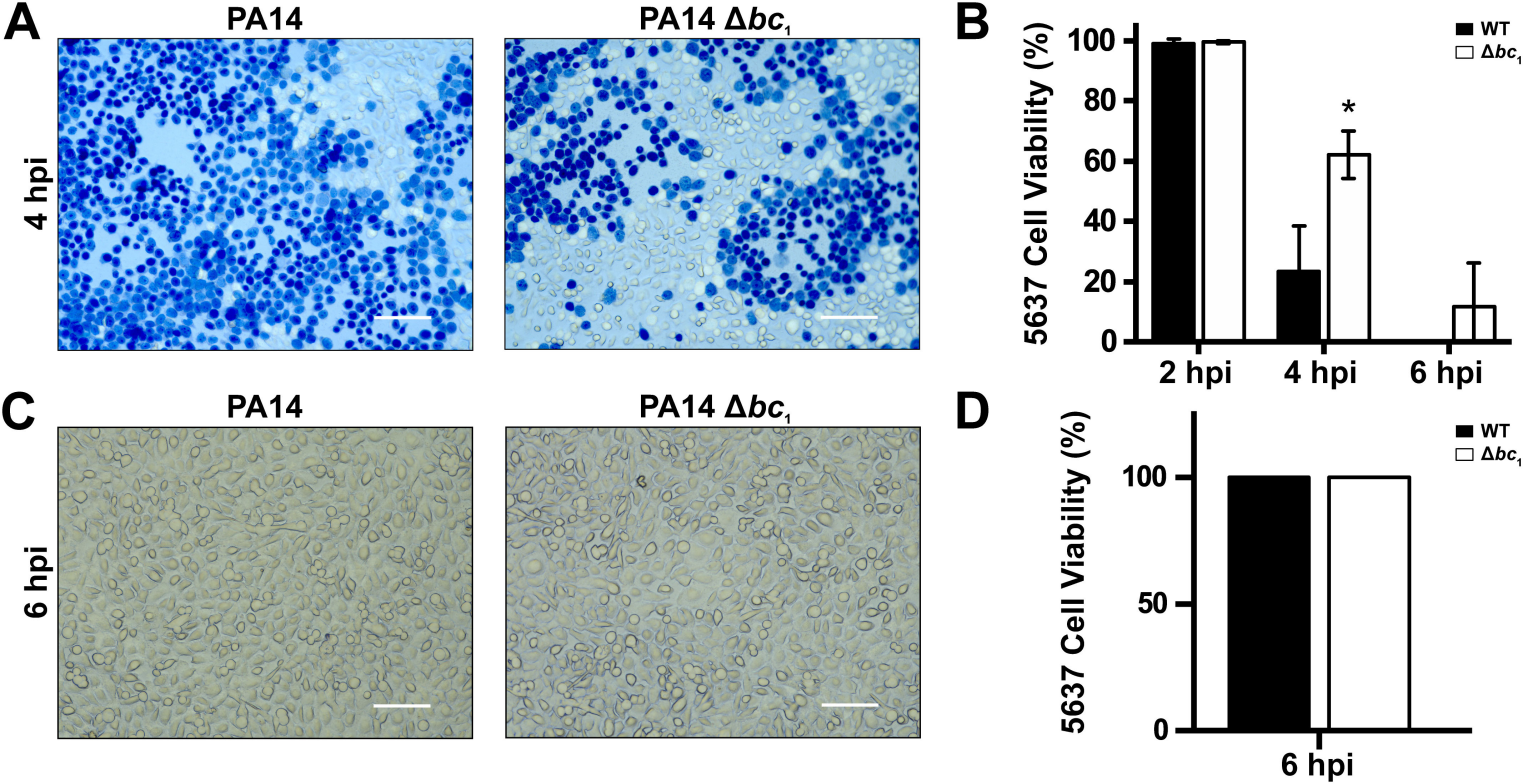
Human bladder epithelial cell viability following infection with PA14 or PA14 Δ*bc*_1._**(A)** Representative images of 5637 cells infected with PA14 or PA14 Δ*bc*_1_ at a multiplicity of infection of 10 for 4 hours and stained with 1:1 PBS:trypan blue, followed by microscopy imaging. Scale bar = 100 µm. 5637 viability was quantified as percentage of live cells (unstained) versus all cells [unstained plus blue-stained (dead)] of 10 different random fields at 2, 4, and 6 hpi **(B)**. Results are presented as mean ± SD, *n* = 3. Cell culture supernatant following 6 hours of infection with PA14 or PA14 Δ*bc*_1_ was filtered and reapplied to 5637 bladder epithelial cells and incubated for 6 hours. **(C)** Representative images of 5637 cells infected with filtered supernatant of PA14 and PA14 Δ*bc*_1_ stained with 1:1 PBS:trypan blue, and imaged via microscopy. Viability of cells infected with filtered supernatant was quantified as percentage of live cells (non-stained) versus all cells [non-stained plus blue-stained (dead)] of 10 different random fields and reported as mean ± SD, *n* = 3 **(D)**. Statistical differences between PA14 and PA14 Δ*bc*_1_ were evaluated via *t*-test analysis (*p < 0.05).

To characterize the role of cytochrome *bc*_1_ in *P. aeruginosa* virulence and pathogenicity, 5637 human epithelial bladder cells were infected with either wild-type or Δ*bc*_1_ mutant strains, and bladder cell viability was assessed (**Figure 2B**). At 2 hpi, neither strain impacted bladder cell survival, indicating that this timeframe is necessary for the bacterial cells to express virulence factors. At 4 hpi, the viability of cells infected with wild-type *P. aeruginosa* drops sharply to 23% (**Figures 2A, B**). On the other hand, 62% of cells infected with the mutant strain are still viable (**Figures 2A, B**). By the 6-hour time point, the wild-type strain eliminated all viable human cells, while in the mutant 12% of the infected cells are still viable. Even though cellular viability decreases over time in both conditions, virulence is clearly diminished by the lack of cytochrome *bc*_1_, which may be due to alterations in bacterial virulence factor production. Thus, we sought to investigate how the mutation attenuates pathogenicity during host cell infection. Following 6-hour bladder cell infection with PA14 or PA14 Δ*bc*_1_ at MOI 10, culture media was collected, filtered (0.2 µm), and applied to uninfected bladder epithelial cells for 6 hours. Subsequent evaluation of cellular viability shows that neither wild-type nor Δ*bc*_1_ mutant (**Figures 2C, D**) produce soluble factors that induce bladder cell death in the conditions tested, as the bladder cells exposed to the filtered media retained 100% cell viability (**Figure 2D**). This suggests that the virulence factor(s) downregulated in the mutant requires cell-to-cell contact-mediated mechanisms.

### Cytochrome *bc*_1_ is essential for T3SS functionality

**Figure 3:**
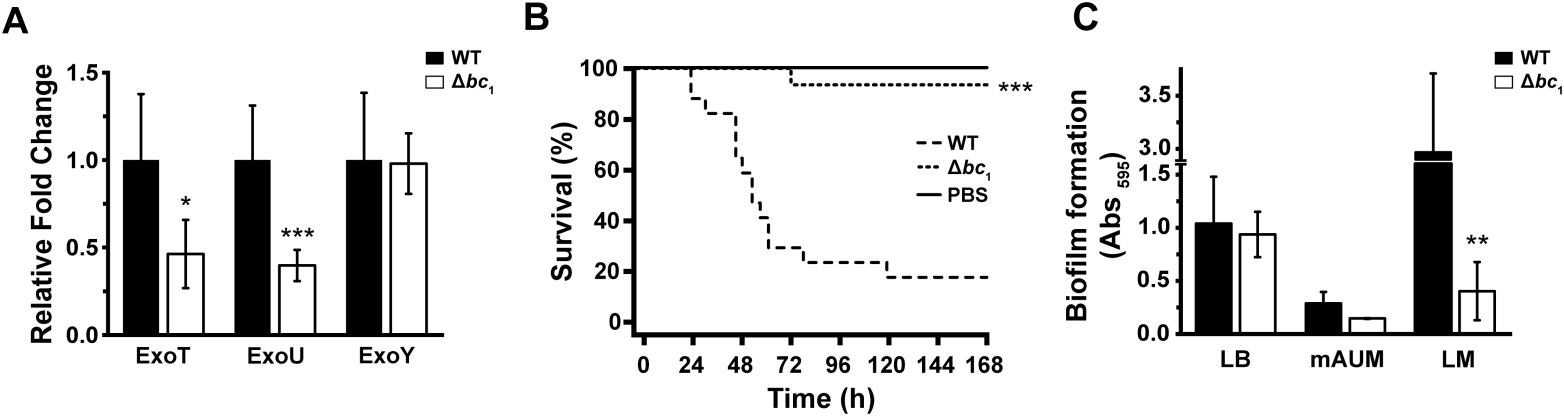
Effects of cytochrome *bc*_1_ deletion on *P. aeruginosa* virulence. 5637 cells were infected with PA14 or PA14 Δ*bc*_1_ for 4 hours at a multiplicity of infection of 10. **(A)** Relative mRNA expression of the T3SS effectors *exoT*, *exoU*, and *exoY* was subsequently quantified via qPCR. Results are presented as mean ± SE, *n* = 9. **(B)** Kaplan-Meier survival curves of eight-week-old female BALB/cJ mice following systemic infection with PA14 (*n* = 17) or PA14 Δ*bc*_1_ (*n* = 14) administered through lateral tail vein. PBS was used as a vehicle control (*n* = 7). **(C)** Biofilm production by PA14 or PA14 Δ*bc*_1_ in LB, mAUM, and LM following 48 hour incubation (n = 3). Statistical significance between PA14 and PA14 Δ*bc*_1_ was determined by *t*-test (*p < 0.05, **p < 0.01, ***p < 0.001). Mouse survival significance was evaluated using the log-rank test (***p < 0.001).

*P. aeruginosa* possesses a type III secretion system (T3SS) that is critical for contact-based cytotoxicity, enabling the injection of proteins into the host cell cytoplasm (Hauser, 2009; Stover et al., 2000). PA14 secretes exoT, exoU, and exoY effector proteins, which function distinctly yet complementarily throughout the infection process (Filloux, 2011). ExoU is the major virulence factor *in vivo* and *in vitro* due to its phospholipase activity, which compromises the host membrane, while exoT and exoY have been shown to play a minimal role during infection (Hauser, 2009; Kazmierczak and Engel, 2002; Krall et al., 2000; Lee et al., 2005; Phillips et al., 2003; Sato et al., 2003). Relative fold-change analysis of T3SS effector gene expression (**Figure 3A**) showed a 53% decrease in *exoT* and a 60% decrease in *exoU* expression in the deletion mutant strain. However, *exoY* expression remained unchanged between the strains. Interestingly, it was previously reported that *P. aeruginosa* strains lacking the gene encoding for *exoY* or failing to express the protein were more toxic to lung epithelial cells than strains expressing the toxin (Silistre et al., 2021), indicating that *exoY* presence is not detrimental to host cells. These results demonstrate that deletion of cytochrome *bc*_1_ severely hinders *P. aeruginosa* virulence (**Figures 2B**, **3A**) by downregulating expression of the most cytotoxic effectors *exoT* and *exoU*, plausibly due to its compromised respiratory chain function and thus bioenergetic processes. The *P. aeruginosa* T3SS needle complex relies on PscN, an ATPase that drives the translocation of effector proteins into host cells (Hauser, 2009). Deletion of cytochrome *bc*_1_ in PA14 disrupts oxidative phosphorylation and diminishes cellular ATP generation (see below), thereby limiting the energy supply required for T3SS function. As a result, the molecular syringe may operate less efficiently, leading to reduced effector protein secretion and attenuated host cell damage.

### Deletion of the *bc*_1_ complex decreases virulence *in vivo*

To further evaluate the impact of cytochrome *bc*_1_ deletion on *P. aeruginosa* pathogenicity, the effects of the mutant were assessed on a systemic infection model in mice, evaluating survival following infection with wild-type or Δ*bc*_1_ cells. As shown in **Figure 3B**, <20% of mice infected with wild-type PA14 survived beyond seven days post-infection. Remarkably, deletion of cytochrome *bc*_1_ increased survival to 93%, indicating that this enzyme is essential for virulence *in vivo*. The deletion mutant is completely avirulent, as the survivability did not differ statistically from that of the PBS control group (**Figure 3B**). This effect may be attributed to the bacterium’s energy deficit paired with lack of virulence factor production, resulting in an inability to resist host immune response. Indeed, *exoU* has been associated with phagocyte killing (Diaz et al., 2008), thereby compromising host defense mechanisms. Consequently, diminished *exoU* expression in the Δ*bc*_1_ mutant strain may mitigate virulence and would promote bacterial clearance in mice.

### Role of the *bc*_1_ complex in biofilm formation

Biofilm is a critical virulence factor of *P. aeruginosa*, contributing to both infection establishment and multidrug resistance in different tissues, including the lung and urinary tract (Maurice et al., 2018). In this work, we further characterized the role of the *bc*_1_ complex in pathogenicity by evaluating biofilm formation in physiologically relevant media. As shown in **Figures 3C**, in LB medium, both wild-type and mutant strains produce comparable amounts of biofilm. In contrast, a significant difference between the wild-type and mutant is observed in LM, where the mutant had a seven-fold decrease in biofilm formation. Interestingly, in mAUM, the cells could not produce significant amounts of biofilm. These findings underscore the critical role of the *bc*_1_ complex in facilitating biofilm formation in LM, suggesting that the enzyme may serve as a promising therapeutic target for disrupting *P. aeruginosa* in biofilm-associated infections, which may be particularly relevant in lung infections, in conditions such as cystic fibrosis.

### Role of the *bc*_1_ complex in ATP generation, reactive oxygen species production, and membrane potential in physiologically relevant media

**Figure 4:**
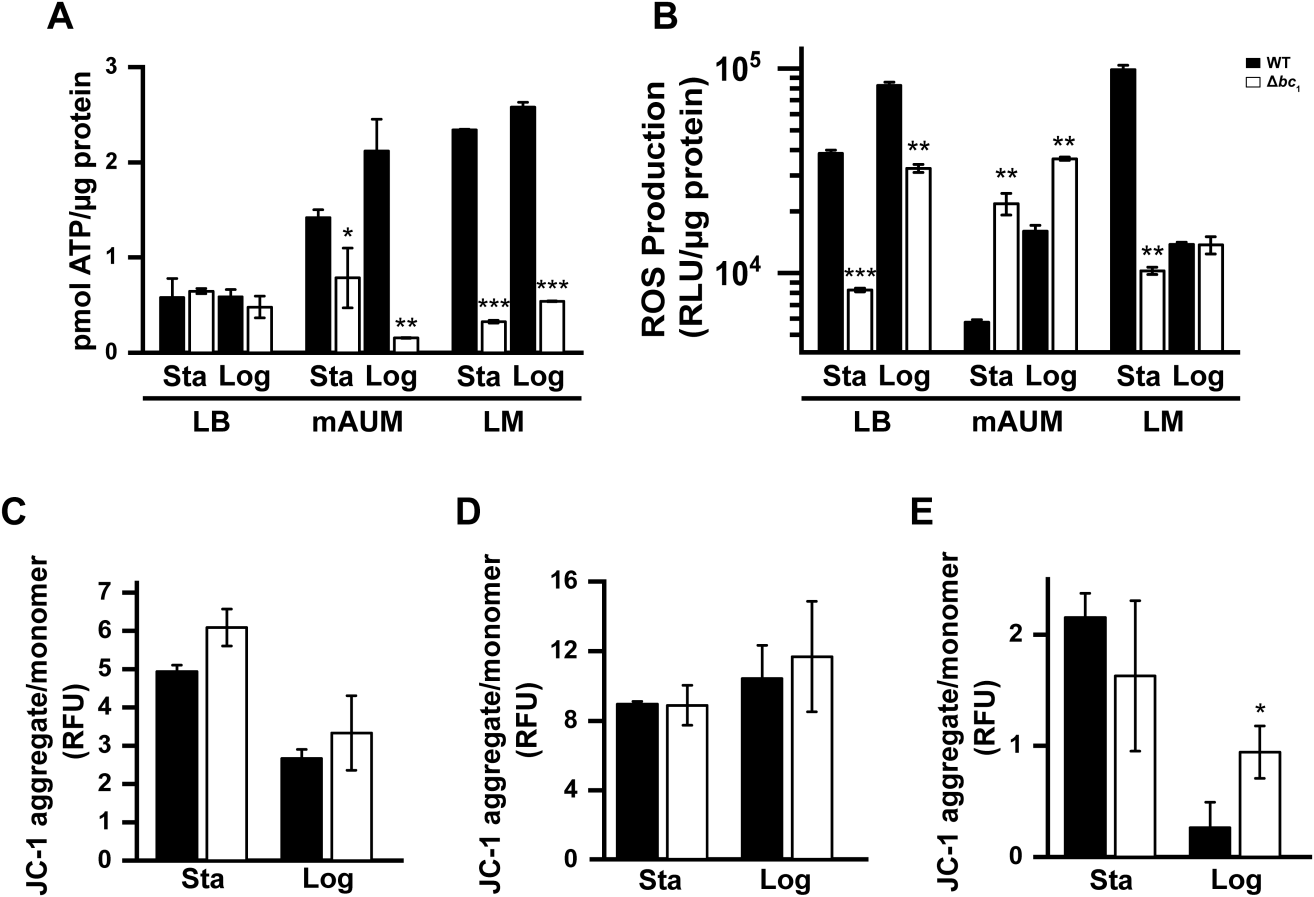
Assessment of PA14 and PA14 Δ*bc*_1_ bioenergetics in physiologically relevant medium during stationary and logarithmic phases. ATP levels **(A)**, ROS production **(B)**, and membrane potential in LB **(C)**, mAUM **(D)**, and LM **(E)** were evaluated in PA14 and PA14 Δ*bc*_1_ in stationary (Sta) and logarithmic (Log) phases. Results are reported as mean ± SD, *n* = 3. Statistical differences between PA14 and PA14 Δ*bc*_1_ were analyzed via *t*-test (*p < 0.05, **p < 0.01, ***p < 0.001).

As shown in our previous studies, the electron transport chain is essential for ATP generation in *P. aeruginosa*, contributing to the formation of the proton gradient, which drives energy production (Mitchell, 2011). ATP levels of PA14 and PA14 Δ*bc*_1_ were measured in cells cultured in LB, mAUM, and LM to determine the relevance of cytochrome *bc*_1_ in ATP generation under physiologically relevant conditions (**Figure 4A**). In laboratory LB media, no difference was found in ATP content between the wild-type strain and mutant in stationary or logarithmic phases, which is consistent with the minor changes in growth found in this condition. However, in mAUM, the ATP content of the Δ*bc*_1_ mutant was 44% less than the wild-type strain during stationary growth. This effect was more pronounced during the logarithmic phase of growth in mAUM, as the mutant produced 92% less ATP than the wild-type strain. In lung media, ATP content was severely decreased in the Δ*bc*_1_ mutant in both stationary and logarithmic phases, by 86% and 79% compared to the wild-type strain, respectively. These results clearly demonstrate that the *bc*_1_ complex, while not absolutely essential for growth, is critical in *P. aeruginosa* bioenergetics and physiology. Remarkably, the effects of the mutant on ATP content closely correlate with the effects seen in the growth parameters under physiologic culture conditions as shown in **Figure 1**. Altogether, the data indicate that cytochrome *bc*_1_ is highly relevant to energy generation in physiologic media, which explains the almost complete lack of virulence of the mutant in animal models.

As reported in the literature, cytochrome *bc*_1_ is one of the main contributors to reactive oxygen species production (Brand, 2016; Seaver and Imlay, 2004). To fully characterize the metabolic role of the *bc*_1_ complex in *P. aeruginosa*, ROS production was evaluated in the mutant and wild-type strain. Our results show that ROS production was significantly modified in the mutant strain in all three media tested (**Figure 4B**). In LB medium during stationary and logarithmic stages of growth, ROS production by PA14 Δ*bc*_1_ significantly decreased compared to the wild-type strain, by 78% and 60%, respectively. In LM, there was no difference between the two strains during logarithmic growth, while a nearly 10-fold decrease in ROS production was observed in the mutant strain during the stationary phase. The decrease in ROS production by the mutant in these conditions is expected, as the *bc*_1_ complex is a main contributing factor to oxygen radicals (Pagacz et al., 2021). Surprisingly, in mAUM, ROS production by the mutant strain increased significantly compared with PA14, showing a 3.8- and 2.2-fold increase in stationary and logarithmic growth phases, respectively. This increase likely reflects the pathogen’s reliance on cytochrome *bc*_1_ in mAUM, where it drives ion pumping and respiration (Hu et al., 2024). The lack of the *Pseudomonas* quinolone signal (PQS) in the mutant (Soh et al., 2021), which is linked to the generation of anti-oxidative responses (Häussler and Becker, 2008), may also contribute to this surge in ROS in mAUM.

As discussed above, our previous work shows that cytochrome *bc*_1_ is a critical contributor to ion pumping in the *P. aeruginosa* respiratory chain (Hu et al., 2024), which links the operation of the respiratory chain to ATP production. To evaluate the role of cytochrome *bc*_1_ on membrane potential, the fluorescent signal of the voltage-dependent JC-1 dye was studied in the wild-type and Δ*bc*_1_ mutant in LB, mAUM, and LM (**Figures 4C-E**). The addition of oligomycin produced relatively small changes in membrane potential and thus the data were omitted from this work for clarity. In LB, wild-type cells produced a significant membrane potential in the stationary phase, which decreased in the logarithmic phase. In this condition, no significant differences were found in the Δ*bc*_1_ mutant (**Figure 4C**), which correlates with the small differences observed in growth and ATP content. This effect could be due to the significant role that other respiratory enzymes, such as NQR, *bd* oxidase, and *bo*_3_ oxidase, play in membrane potential generation, which could compensate for lack of the *bc*_1_ complex, as discussed in a previous work (Hu et al., 2024). In mAUM (**Figure 4D**), the membrane potential produced by wild-type cells during stationary phase nearly doubled compared to that of LB, and did not decrease in the logarithmic state, which could be due to the activation of the respiratory chain observed in these conditions (Hu et al., 2024). Remarkably, in mAUM, the Δ*bc*_1_ mutant did not exhibit a difference in membrane potential compared to the wild-type strain. Finally, membrane potential measurements for cells cultured in LM were carried out in fresh LM instead of PBS, which was used for other conditions, as LM-grown cells displayed an undetectable membrane potential when measured in PBS. The data shown in **Figure 4E** indicate that in this condition, the cells produce a small but detectable membrane potential, which was significantly increased in the mutant during logarithmic growth. Experiments were also carried out with DiOC_2_(3), another widely used membrane potential indicator (Novo et al., 1999; Pan et al., 2017), obtaining similar results (not shown). It is likely that the nutrient composition of LM does not allow for a very active metabolism, reflected in the lowest growth rate of all conditions (approximately 20% compared to LB medium). In spite of the significant changes in the metabolic parameters that we have measured, including ATP content, in the case of membrane potential, the mutant only showed a significant increase compared to the wild-type strain during logarithmic growth in LM, which indicates that the respiratory chain in the mutant is adapted to the lack of the *bc*_1_ complex in most conditions, likely by decreasing the expression of pathways that require active H^+^- transport, which would result in diminished growth and an avirulent phenotype.

### Metabolic adaptations of the cytochrome *bc*_1_ mutant

**Figure 5:**
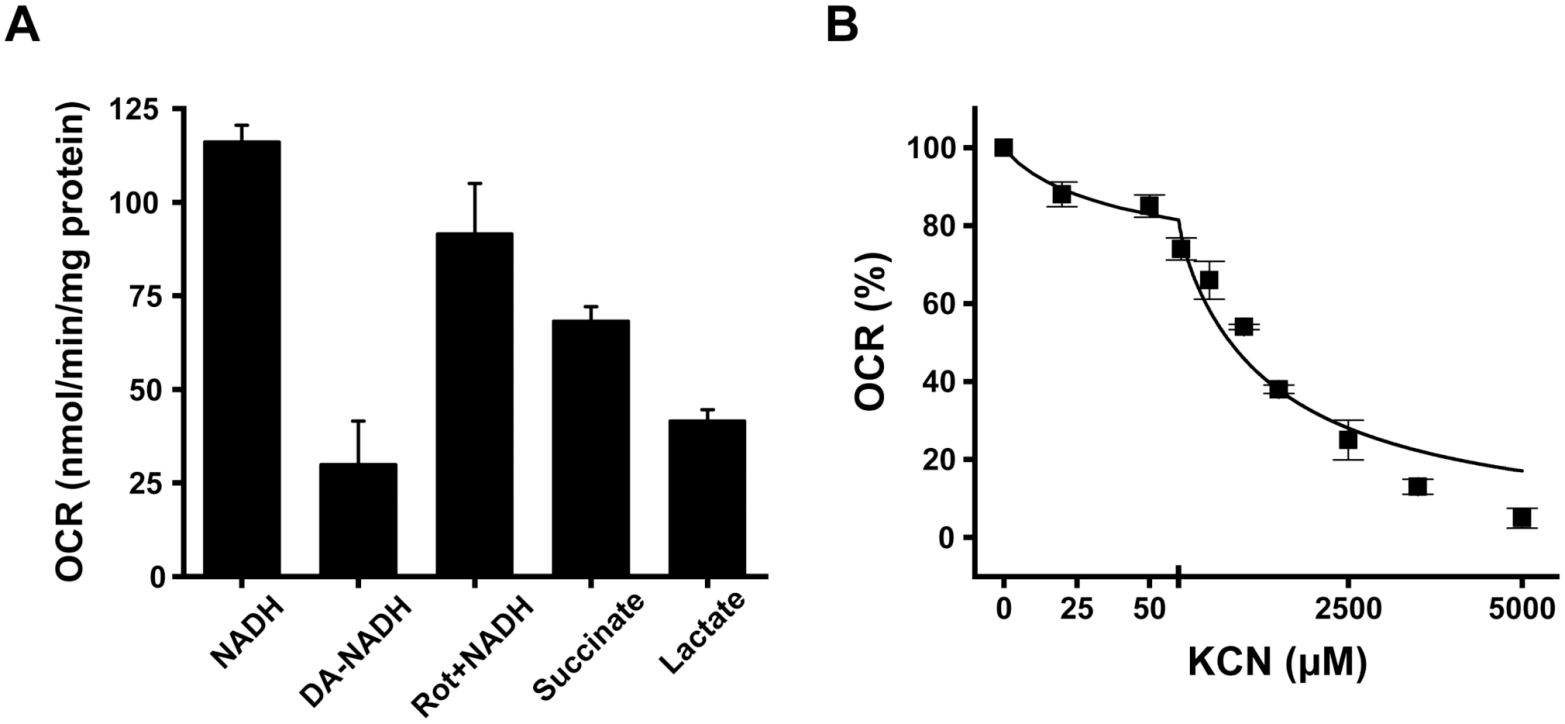
Metabolic adaptations of PA14 Δ*bc*_1_. Oxygen consumption rates of PA14 Δ*bc*_1_ membranes harvested during the logarithmic phase of growth in mAUM were quantified in the presence of different substrates (200 µM NADH, 200 µM deamino-NADH, 1 µM rotenone, 1 mM succinate, 10 mM lactate) **(A)**. Ubiquinol oxidase contribution was assessed via quantification of oxygen consumption rates at various concentrations of KCN with 50 µM ubiquinone-1 and 500 µM 1,4-Dithiothreitol (DTT) **(B)**. All results are reported as mean ± SD, *n* = 3.

As shown in this manuscript and our previous work, the *bc*_1_ complex has a critical role in cell physiology. Nonetheless, this enzyme is not essential for *P. aeruginosa*, as the cells are able to adapt to its elimination and can grow in all physiologic media tested, although at lower rates. However, the significant metabolic challenge imposed by this deletion has a deleterious effect on virulence. To understand the adaptability of *P. aeruginosa*, the respiratory chain of the mutant was fully characterized as previously described (Hu et al., 2024; Liang et al., 2020). *P. aeruginosa* has three NADH:ubiquinone oxidoreductases: Complex I, NQR, and NDH-2, which are not homologous and can be distinguished by their substrate and inhibitor sensitivity (Degli Esposti, 1998; Matsushita et al., 1987; Yagi, 1991; Zambrano and Kolter, 1993; Zhou et al., 1999; Zickermann et al., 2000). NQR and complex I, which are proton pumps (Raba et al., 2018), are capable of utilizing both NADH and deamino-NADH (Matsushita et al., 1987; Zhou et al., 1999). Complex I is able to do so without showing substrate preference, and is inhibited by rotenone (Degli Esposti, 1998; Zickermann et al., 2000), while NQR is rotenone insensitive (Zhou et al., 1999). In contrast, NDH-2, which does not have proton pumping activity, specifically uses NADH as a substrate and is resistant to rotenone (Matsushita et al., 1987; Yagi, 1991; Zambrano and Kolter, 1993). Thus, the activities of these enzymes can be differentiated by measuring the respiratory activity with NADH, deamino-NADH, and rotenone, as showed previously by our group (Liang et al., 2020). **Figure 5A** depicts the oxygen consumption rate (OCR) of *P. aeruginosa* Δ*bc*_1_ membranes harvested in mAUM during the logarithmic growth stage using NADH, deamino-NADH, succinate, and lactate as substrates. These substrates are employed by the main dehydrogenases involved in the pathogen’s respiratory chain in mAUM, contributing to the ubiquinone/ubiquinol pool that can ultimately be utilized by the terminal oxidases (Hu et al., 2024; Liang et al., 2020). In comparison to the succinate and lactate dehydrogenase activities, the NADH oxidase activity in the mutant exhibits the highest OCR, indicating its critical role in respiration. NADH activity was mostly insensitive to rotenone, indicating that complex I is minimally involved, facilitating approximately 20% of electron transfer. Moreover, deamino-NADH oxidation was relatively lower compared to the wild-type cells (Hu et al., 2024; Liang et al., 2020), revealing that NQR and complex I contribute <35% of the NADH dehydrogenase activity in the cytochrome *bc*_1_ mutant, and that the mutant mainly relies on NDH-2, supporting around 65% of electron transfer from NADH. This result contrasts sharply with wild-type *P. aeruginosa*, in which 75% of the NADH dehydrogenase activity is provided by NQR (Hu et al., 2024; Liang et al., 2020).

To assess the respiratory contribution of terminal oxidases in the cytochrome *bc*_1_ mutant, respiratory ubiquinol oxidase relative activities were analyzed through KCN titration, as previously described (**Figure 5B**) (González-Montalvo et al., 2024; Hu et al., 2024; Liang et al., 2020). In addition to the *bc*_1_ complex, which feeds cytochrome *c* oxidases, the respiratory chain of *P. aeruginosa* contains two ubiquinol oxidases: cytochrome *bo*_3_ oxidase (Arai, 2011), which is sensitive to cyanide in the 10-30 μM range (Liang et al., 2020), and the cyanide-insensitive *bd* oxidase, which can be inhibited in the mM range (Arai et al., 2014; Liang et al., 2020). In logarithmic phase cells cultured in mAUM, the primary oxidase employed by the Δ*bc*_1_ mutant is the cyanide-insensitive *bd* oxidase, carrying out nearly 70% of the activity, while the remaining 30% can be attributed to *bo*_3_ oxidase. According to our data, the respiratory chain in the wild-type is composed of NQR and *bc*_1_ in addition to cytochrome oxidases, and in the mutant it switches to NDH-2 and *bd* oxidase, which significantly reduces the ability of the respiratory chain to pump protons and produce ATP (see below).

## Discussion

Infections caused by *P. aeruginosa* in the urinary tract and lungs pose a major healthcare challenge, largely due to the pathogen’s MDR profile (Reynolds and Kollef, 2021). Despite its clinical importance and impact, the metabolic strategies that enable *P. aeruginosa* to colonize distinct physiologic environments and contribute to virulence remain poorly understood. Prior research has revealed elevated activity of the Krebs and glyoxylate cycles in *P. aeruginosa* in both chronic lung infections (Hoboth et al., 2009) and urinary tract infections (Berger et al., 2014), highlighting the importance of aerobic metabolism during infection. Our group previously examined the aerobic metabolism of *P. aeruginosa* in media resembling urine, mAUM (Hu et al., 2024; Liang et al., 2020), identifying cytochrome *bc*_1_ as the major contributor to respiration and ion pumping, which indicated its potential as an antibiotic target (Hu et al., 2024). Cytochrome *bc*_1_ inactivation by transposon mutagenesis previously showed a reduction in virulence factor production and pathogenicity of *P. aeruginosa* PAO1 (Shen et al., 2021). However, a thorough investigation of the mechanism by which the *bc*_1_ complex regulates homeostasis and virulence has not been carried out, particularly in the PA14 strain, which is relevant for clinical cases due to its high pathogenicity. In this manuscript, we have expanded our studies to fully understand the role of this enzyme in *P. aeruginosa* physiology in critical infection sites: the lungs and the urinary tract.

### Cytochrome *bc*_1_ is critical for *P. aeruginosa* growth in urinary- and lung-like environments

In our previous work, we reported that the growth of a PA14 cytochrome *bc*_1_ transposon mutant (Liberati et al., 2006) showed significant defects in maximum growth and growth rate, and an increase in the lag phase (Hu et al., 2024). In this study, we obtained a deletion mutant of cytochrome *bc*_1_ in PA14 using the lambda-red recombinase method (Datsenko and Wanner, 2000), which specifically eliminates the gene of interest and minimizes unintended effects of transposon mutagenesis, which can inactivate neighboring genes or regulatory sequences (Datsenko and Wanner, 2000). Moreover, we expanded our studies to LM (Ruhluel et al., 2022) to understand the effects of the mutant in the highly relevant lung environment, particularly for opportunistic infections in cystic fibrosis. Our results show significant growth defects in the mutant, including an increase in the lag phase (**Figure 1F**) and decreases in the growth rate (**Figure 1E**) and yield (**Figure 1D**) in rich laboratory LB media (**Figure 1A**), which were considerably more pronounced in physiologic media. The differences observed among culture media may be due to their specific composition. For instance, mAUM contains lactate and citrate as main carbon sources (González-Montalvo et al., 2024; Liang et al., 2020), while LM is composed mostly of amino acids (Ruhluel et al., 2022). In both mAUM and LM, an active Krebs cycle and respiratory chain are required for growth, underscoring the relevance of the *bc*_1_ complex in these conditions due to its involvement in electron transport. In mAUM, *P. aeruginosa* relies on a tightly coupled, oxidative TCA cycle with strong gluconeogenic flux and high energetic efficiency, which could be related to faster growth. In contrast, in LM the metabolism becomes flexible and nitrogen-driven, with amino acid catabolism feeding multiple TCA entry points. In our previous work, the relative contribution of respiratory enzymes to ATP production was calculated, demonstrating that the *bc*_1_ complex is the main respiratory enzyme in mAUM, contributing more than 50% of the respiratory chain H^+^-pumping activity (Hu et al., 2024). The relevance of the *bc*_1_ complex in *P. aeruginosa* is confirmed in this work by the slow growth of the mutant in mAUM (**Figures 1B, E**). Remarkably, our data show that the *bc*_1_ complex is also critical for proliferation in the lung-mimicking environment, where a sharp reduction in growth was observed in the mutant in comparison to the wild-type strain (**Figures 1C, E**). Relative to mAUM, the wild-type strain grew to higher OD_620_ in LM but exhibited a lower growth rate. In the mutant, we observed an extended lag phase, indicating that cytochrome *bc*_1_ plays important roles in adaptability to nutritional sources, particularly to the amino acid-rich lung media, which mirrors the conditions typically observed in cystic fibrosis lungs (Barth and Pitt, 1996). Amino acid catabolism feeds directly into the Krebs cycle, generating reducing equivalents that drive the electron transport chain. Thus, in an amino acid-rich medium such as LM, the cytochrome *bc*_1_ mutation is expected to produce a significant growth defect.

Biofilms are hallmark contributors to the persistence of chronic urinary and pulmonary infections and are an important virulence factor of *P. aeruginosa* (Folkesson et al., 2012; Mancuso et al., 2023). Our results show that in LB, the wild-type strain was able to produce a significant amount of biofilm, which was not affected by the lack of the *bc*_1_ complex. On the other hand, in mAUM, PA14 produced moderate amounts of biofilm, approximately 28% compared to the LB condition. Interestingly, in mAUM, the Δ*bc*_1_ mutant showed a decrease of 50% in biofilm production compared to the wild-type (**Figure 3C**). Indeed, a previous report indicates that in urea-rich environments, such as those in the urinary tract and mAUM, quorum sensing is repressed in *P. aeruginosa* (Cole et al., 2018), which plays an essential role in biofilm development (Miranda et al., 2022). In LM, the wild-type strain showed nearly a 3-fold increase in biofilm production compared to LB that could be due to high mucin content in LM, which may aid in bacterial attachment and aggregation, mimicking cystic fibrosis environments (Landry et al., 2006). Remarkably, biofilm production was severely diminished in the mutant strain (seven-fold reduction). The attenuation in biofilm production by the Δ*bc*_1_ mutant in physiologic media can be attributed to a decrease in electron flow through cytochrome *bc*_1_, which diminishes both aerobic respiration via terminal cytochrome *c* oxidases and anaerobic respiration via denitrification. Several reports indicate that in the lung environment, especially during cystic fibrosis and in biofilms, *P. aeruginosa* switches its metabolism and is able to generate energy anaerobically via denitrification (Hassett et al., 2002; Ojoo et al., 2005; Williams et al., 2007), which employs nitrate and nitrite reductases dependent on cytochrome *bc*_1_ activity (Arai, 2011; Williams et al., 2007). A previous report also demonstrates that inactivation of the cytochrome *c*_1_ subunit of cytochrome *bc*_1_ halted *P. aeruginosa* growth in anaerobic conditions using nitrite as an electron acceptor (Hasegawa et al., 2003). Alternatively, although the arginine deiminase (ADI) pathway is also utilized by *P. aeruginosa* under conditions when oxygen and nitrate are not present (Williams et al., 2007), increased expression of key ADI enzymes has been observed in CF lungs, indicating that this pathway is also employed during such infections (Hogardt and Heesemann, 2013). However, our data demonstrate that denitrification via the cytochrome *bc*_1_-dependent respiratory chain is the predominant pathway supporting energy generation in *P. aeruginosa* biofilms under the tested conditions, as exemplified by the decreased biofilm produced by the mutant. As biofilm formation is a key virulence factor in *P. aeruginosa* in both pulmonary and urinary tract infections, reducing biofilm is critical for decreasing persistence, reducing colonization, and enhancing antibiotic efficacy (Narten et al., 2012; Ciofu et al., 2017; Soares et al., 2020). By identifying the metabolic relevance of cytochrome *bc*_1_ for *P. aeruginosa* biofilm, we have demonstrated that therapeutic targeting of cytochrome *bc*_1_ holds promise, particularly in the pulmonary environment of cystic fibrosis patients, which may also enhance current antibiotic efficacy via synergistic interactions. Our data and previous findings highlight the potential of cytochrome *bc*_1_ as an antibiotic target that can be exploited in both aerobic and anaerobic conditions.

### Role of cytochrome *bc*_1_ in virulence regulation

Our data show that deletion of cytochrome *bc*_1_ reduces *P. aeruginosa* growth in physiologic conditions and decreases the virulence of cells in an *in vitro* infection model of 5637 human urinary bladder carcinoma cells, a system that recapitulates part of the urinary tract infection. In these conditions, the mutant has a significant attenuation of virulence, as there is a 39% difference between the death of Δ*bc*_1_-infected and PA14-infected cells (**Figures 2A, B**), which is not mediated by the secretion of virulence factors, but is contact-based (**Figures 2C, D**). qPCR experiments show that the mutant decreases expression of the essential T3SS components *exoT* and *exoU*, while *exoY* remains unchanged (**Figure 3A**). *ExoU* is encoded on the PAPI-2 pathogenicity island in PA14, which contains other non-*exoU* virulence genes (Harrison et al., 2010). The decrease of *exoU* expression in the mutant strain suggests that other virulence genes encoded within PAPI-2 may also have downregulated expression. Moreover, previous findings revealed that the respiratory chain is critical for *P. aeruginosa* pathogenicity in a *Galleria mellonella* model (Torres et al., 2019). Prior reports also show that the elimination of the *bc*_1_ complex decreases *P. aeruginosa* virulence factor production and hinders motility (Shen et al., 2021). While these reports show a similar trend, the mechanism of *bc*_1_ virulence regulation has remained unclear until now. In this work, we have shown that elimination of the *bc*_1_ complex reduces ATP content by 44-92%, which can explain the reduced virulence, as the T3SS is a molecular motor that requires ATP for its function (Hauser, 2009). Thus, factors that affect energy production should have a direct effect on contact-based virulence. In this report, we extended the studies carried out on invertebrate models by investigating the role of cytochrome *bc*_1_ in the virulence of *P. aeruginosa* in a clinically-relevant *in vivo* systemic murine infection model. Our results show that while the wild-type strain is highly virulent, producing >80% mortality in less than a week, the PA14 Δ*bc*_1_ strain is completely avirulent, demonstrating the critical role of the enzyme’s contribution to *P. aeruginosa* pathogenicity (**Figure 3B**).

It has previously been suggested that the highly branched respiratory chain of *P. aeruginosa*, in particular the diversity of terminal oxidases, is advantageous for pathogenicity (Hirai et al., 2016; Jo et al., 2014) and survival in different environments, such as biofilms, in which oxygen gradients are established (Jo et al., 2022). However, our data challenges this hypothesis and shows that cytochrome *bc*_1_ is the most important respiratory enzyme in most conditions, including planktonic growth in physiologic media, and in biofilms. The critical role of the *bc*_1_ complex is likely due to its involvement in supporting the activity of the terminal cytochrome *c* oxidases: *caa_3_*, *cbb_3_*-1, and *cbb_3_*-2, which contribute the greatest amount of usable energy for the pathogen under aerobic conditions (Williams et al., 2007; Liang et al., 2020; Srivastav et al., 2025). Indeed, cytochrome *c* oxidases have been reported as essential for *P. aeruginosa* PA14 biofilm and pathogenicity in *Caenorhabditis elegans* (Jo et al., 2017), as deletion of subunit CcoN4, which makes complexes with *cbb_3_–*1 and *cbb_3_*-2 (Hirai et al., 2016), impairs biofilm architecture and improves nematode survival (Jo et al., 2017). Furthermore, elimination of *bc*_1_’s Rieske subunit gene has been reported to interfere with the pathogen’s PQS, hindering biofilm formation (Soh et al., 2021). In addition to its critical role in aerobic conditions, the *bc*_1_ complex also has a fundamental role under anaerobic environments, as it supports cytochrome *c* reduction, which is required for nitrate and nitrite-based anaerobic respiration (Williams et al., 2007). Taken together, the data strongly support a critical role of the *bc*_1_ complex in virulence, supporting the bioenergetic pathways of the cell necessary for the survival of the pathogen or the expression or secretion of virulence factors.

### Cytochrome *bc*_1_ plays critical bioenergetic roles under physiologic conditions

Our data show that in both physiologic conditions, mAUM and LM, the ATP content of wild-type PA14 cells increased 2.4-4.3 times compared to LB, which suggests that the respiratory metabolism is activated. Indeed, we reported that cells grown in mAUM show an increase of about 3 times in the respiratory activity (Hu et al., 2024), consistent with the observed increase in ATP content in this condition (**Figure 4A**). Based on this result, we propose that the respiratory activity of cells grown in LM could also be activated and that the *bc*_1_ complex would be even more important in this condition. Our results show that the elimination of cytochrome *bc*_1_ significantly decreased ATP in the physiologically relevant mAUM and LM media in both growth phases. These results independently corroborate the hypothesis proposed by our group that electron transfer through cytochrome *bc*_1_ (and the associated terminal oxidases) is the most important energy-generating pathway in *P. aeruginosa* (Hu et al., 2024; Liang et al., 2020). This is also consistent with the enzyme’s pumping stoichiometry, as *bc*_1_ and cytochrome *c* oxidases pump 6 protons through the membrane per electron pair (Mitchell, 2011), compared to 2 and 4 protons per electron pair for *bd* (D’mello et al., 1996) and *bo_3_* (Graf et al., 2019) oxidases, respectively (Hu et al., 2024). Interestingly, we found a significant increase in the membrane potential of the mutant compared to the wild-type during logarithmic growth in LM. Membrane potential is a complex metabolic parameter that depends on many factors, including respiratory activity, membrane permeability (regulated by membrane lipid composition), and numerous gradient-consuming processes (ATP synthesis, active transport, motility, etc.). It is likely that due to the limited ability of the respiratory chain to produce a membrane potential, the gradient-consuming steps are significantly downregulated, which would allow the cell to maintain a stable membrane potential but would affect downstream homeostatic processes. However, further experiments are required to clarify this issue.

In addition to the respiratory metabolism, the *bc*_1_ complex would also impact ROS production by the cell (Borisov et al., 2021), as this complex is known to be the main contributor within the electron transport chain (Chance and Hollunger, 1960). Quantification of ROS production demonstrates that the wild-type strain produces significantly more ROS in LB media during both growth phases and LM during stationary phase (**Figure 4B**), consistent with this complex as a major source of ROS. However, in mAUM the mutant produced significantly more ROS compared to the wild-type due to accumulation of electrons in the quinol pool, leading to redirection via the *bd* oxidase. Thus, the excess electrons can react with oxygen, generating superoxide, linking the decreased ATP observed by the mutant in mAUM to the observed increase in ROS. Moreover, other reports have shown that mutants lacking *bc*_1_ do not produce PQS signaling (Soh et al., 2021), which induces an antioxidant response (Häussler and Becker, 2008). This deficiency could account for the increase in ROS production in the mutant strain cultured in mAUM in comparison to the wild-type in both growth phases. During the logarithmic phase in LM, there is no significant difference in ROS production between the wild-type and mutant strains, which can be attributed to both being highly metabolically active during these conditions, prioritizing energy and resources for growth. Furthermore, the extended lag phase and reduced growth rate observed in both strains suggests that ROS mechanisms may be more apparent under non-proliferative or stress-inducing conditions.

### Metabolic adaptations of the cytochrome *bc*_1_ mutant

The data presented here, as well as previous reports in the literature, indicate that the *bc*_1_ complex is critical but not essential for growth, which suggests that the mutant is able to use other metabolic pathways to compensate for its absence. In this report, we show that the mutant compensates for lack of the *bc*_1_ complex by using *bd* oxidase, which intriguingly only pumps 2 H^+^ per electron pair (D’mello et al., 1996), instead of *bo*_3_ oxidase, which pumps twice as many protons (Graf et al., 2019). In addition, the cells shift from NQR, which pumps two cations (Na^+^ or protons) per electron pair (Raba et al., 2018), to NDH-2, which is not a proton pump (Heikal et al., 2014), as the primary NADH dehydrogenase. Thus, the respiratory chain of the mutant (NDH-2 plus *bd* oxidase) pumps a total of 2 protons per electron pair, while the wild-type chain (NQR, *bc*_1_, and cytochrome *c* oxidases) pumps 8 protons per electron pair. Assuming that the metabolic rates through the Krebs cycle are similar in the mutant, this would represent a predicted decrease of 4 times in the ATP production in the *bc*_1_ mutant, which is close to the observed experimental values. Furthermore, our observations regarding growth behavior may also be attributed to lack of ATP production by the mutant, causing the bacteria to compensate by utilizing fermentation or the ADI pathway (Williams et al., 2007). This process does not contribute to formation of the proton gradient, and thus may not be enough to sustain virulence. However, in LB media, we did not see a significant difference in ATP production between the wild-type and mutant strains, which may be due to reliance on fermentation or substrate-level phosphorylation. This drastic decrease in ATP production explains a severe decrease in virulence by the mutant cells *in vitro* and an almost complete lack of virulence in the animal model.

## Conclusions

The findings of this study have deepened our understanding of the metabolic processes employed by *P. aeruginosa* in two physiologically relevant conditions: the lung and urinary tract environments. Our data have demonstrated that cytochrome *bc*_1_ is a promising therapeutic target and that the elimination of this enzyme significantly hinders bacterial growth, virulence, pathogenicity, and energy generation. These results indicate that cytochrome *bc*_1_ plays a fundamental role in the production of the proton gradient, which sustains ATP synthesis (Mitchell, 2011) and all nutrient transport activities. To compensate for the cytochrome *bc*_1_ mutation, *P. aeruginosa* subsequently adapts its metabolism by switching to NDH-2 and *bd*-type terminal oxidase, which support growth but do not sustain a substantial energy metabolism that allows the production of virulence factors. These findings validate cytochrome *bc*_1_ as a possible drug target in *P. aeruginosa* and shed light on the significance of oxidative phosphorylation in virulence and pathogenicity in these diverse environments. Taken together, these data provide valuable insight into potential treatment methodologies that can be implemented to treat this multidrug-resistant pathogen, including targeting the *bc*_1_ complex with antivirulence molecules. Impairing the pathogen’s metabolism through cytochrome *bc*_1_ inhibition would produce a strong reduction in virulence factor production, which may limit disease progression while reducing the selective pressure that drives drug resistance as cytochrome *bc*_1_ is not essential for growth, offering a viable alternative to treat infections produced by this highly relevant pathogen.

## Data Availability

The original contributions presented in the study are included in the article/supplementary material. Further inquiries can be directed to the corresponding author.
